# NH_2_-MIL-125(Ti)/Reduced Graphene Oxide Enhanced Electrochemical Detection of Fenitrothion in Agricultural Products

**DOI:** 10.3390/foods12071534

**Published:** 2023-04-04

**Authors:** Zaixi Shu, Yue Zou, Xuyue Wu, Qi Zhang, Yafang Shen, Anhong Xiao, Shuo Duan, Fuwei Pi, Xiaodan Liu, Jiahua Wang, Huang Dai

**Affiliations:** 1 College of Food Science and Engineering, Wuhan Polytechnic University, Wuhan 430023, China; 2 School of Grain Science and Technology, Jiangsu University of Science and Technology, Zhenjiang 212004, China; 3 Zhejiang Institute of Freshwater Fisheries, Huzhou 313001, China; 4 Food Safety Research Center, Key Research Institute of Humanities and Social Sciences of Hubei Province, Wuhan 430023, China; 5 School of Food Science and Technology, Jiangnan University, Wuxi 214122, China

**Keywords:** fenitrothion, NH_2_-MIL-125(Ti), reduced graphene oxide, electrochemical sensor

## Abstract

The abuse of organophosphate pesticides causes serious threats to human health, which threatens approximately 3 million people and leads to more than 2000 deaths each year. Therefore, it is necessary to determine the residue of fenitrothion (FT) in environmental and food samples. Herein, we developed a non-enzymatic electrochemical sensor with differential pulse voltammetry signal output to determine FT in model solutions and spiked samples. Delicately, the sensor was designed based on the fabrication of hydrothermally synthesized titanium-based metal-organic frameworks (MOFs) material (NH_2_-MIL-125(Ti))/reduced graphene oxide (RGO) (NH_2_-MIL-125(Ti)/RGO) nanocomposites for better target enrichment and electron transfer. The peak response of differential pulse voltammetry for FT under optimized conditions was linear in the range of 0.072–18 μM with the logarithm of concentrations, and the detection limit was 0.0338 μM. The fabricated sensor also demonstrated high stability and reproducibility. Moreover, it exhibited excellent sensing performances for FT in spiked agricultural products. The convenient fabrication method of NH_2_-MIL-125(Ti)/RGO opens up a new approach for the rational design of non-enzymatic detection methods for pesticides.

## 1. Introduction

Pesticides are widely used around the world due to their high efficiency in controlling pests in agriculture to improve the quality and yield of crops [1]. Fenitrothion (FT), also named O,O-dimethyl O-(3-methyl-4-nitrophenyl) phosphorothioate, is a derivative of organophosphorus pesticides and is extensively applied to control insects such as aphids, mites, and whiteflies in cereals, vegetables as well as fruits. However, the abuse of pesticides results in pesticide residues and environmental pollution, which threatens approximately 3 million people and leads to more than 2000 deaths each year [2]. The accumulated FT in contaminated soil and underground water inevitably moves along the food chain and eventually enters the human body. Almost all organophosphorus pesticides irreversibly inhibit acetylcholinesterase activity, leading to the accumulation of acetylcholine and irreversible neurological damage to the human nervous system [3]. Therefore, it is necessary to determine the residue of FT in environmental and food samples.

Up to now, diverse analytical methods, such as chromatographic technique along with mass spectrometry [4,5], surface-enhanced Raman spectroscopy [6], and nuclear magnetic resonance [7], have been developed to determine FT. The above-mentioned analytical methods in centralized laboratories have been successfully used in a wide field of food safety and environmental monitoring. However, they cannot meet the needs of grassroots supervision, market, and household daily inspection owing to the complexity of raw sample preprocessing, expensive instruments, complicated procedures, and other operational constraints. Electrochemical biosensors offer many advantages, including ease-of-use, cost-effectiveness, high sensitivity, and rapid detection, and have become alternative devices for pesticide detection [8,9,10]. Compared to other analytical methods, they can address long analysis time and extensive sample handling, making them popular in FT detection.

Among them, many enzyme inhibition-based electrochemical sensors are developed by measuring the product electroactive thiocholine of the acetylcholinesterase-catalyzed acetylthiocholine reaction, which reflects the presence of organophosphorus pesticides by inhibiting the enzyme activity of acetylcholinesterases [11]. However, these enzyme activity-based methods suffer from enzyme activity, stability, and reactivation, which limit the detection sensitivity and application fields. Therefore, it is a worthy attempt to develop non-enzymatic sensors for the electrochemical detection of FT with high sensitivity, rapidity, and cost-effectiveness. For example, A. Kumaravel employed a nanosilver/dodecane-modified electrode to fabricate a non-enzymatic electrochemical sensor for the determination of FT in potatoes and paddy with a detection limit of 0.60 nM [10]. The conventional bare working electrode had poor detection performance due to its slow electron transfer and poor electrochemical response during FT detection. Current electrochemical sensors typically employ nanomaterials and nanostructures to improve the performance of biosensors [12]. It is a feasible approach to enhance the detection signal through the enrichment of the target on the electrode surface, thus improving the electrochemical detection performance.

Metal-organic frameworks (MOFs) are a set of molecular networks that are arranged on a three-dimensional framework with metal centers and bridging organic ligands [13,14]. Compared with classical solid adsorbents such as zeolites [15], activated carbon [16], and silica materials [17], MOFs have the advantages of adjustable pore size, multiple active sites, and thermal and chemical stability. MOFs have good enrichment properties for effective target enrichment and enhanced signal detection [18,19]. Since curcumin/UiO-66 has strong adsorbability and there is good electrochemical signal amplification from UiO-66, our group loaded curcumin with a metal-organic framework to form a curcumin-UiO-66 composite, and successfully prepared an electrochemical sensor for the direct detection of methyl parathion in fruit and vegetable samples [20]. Nevertheless, the organic structure of MOFs affects electron transfer on bare electrodes, leading to an insufficient effective reaction area of the electrode and low electrical signal intensity [21,22]. Graphene oxide (GO) is a graphite derivative with unique electrical, thermal, and mechanical properties, typically made by chemically oxidizing and scratching graphene to provide a large surface area and abundant adsorption sites [23,24]. Reduced graphene oxide (RGO) is prepared from GO, which has a similar structure to graphene but better electrical conductivity. Thus, the combination of MOFs and RGO in electrochemical sensors is expected to be a good attempt for the detection of FT.

**Scheme 1:**
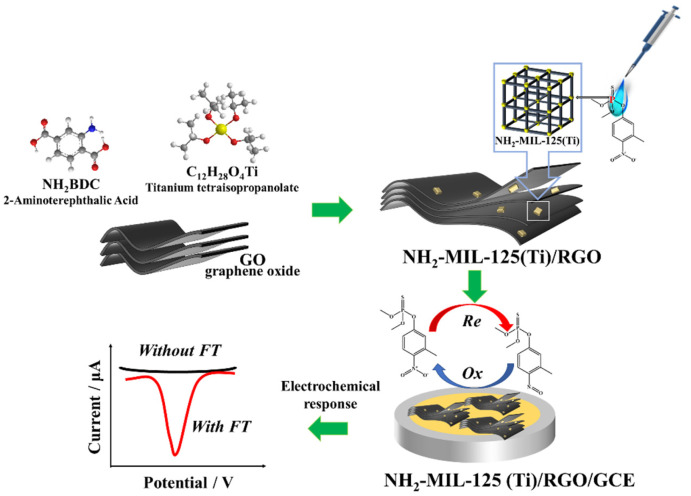
Schematic diagram of materials, NH_2_-MIL-125(Ti)/RGO/GCE preparation process, and detection mechanism.

In this study, NH_2_-MIL-125(Ti)/RGO composites were prepared based on the Material of Institut Lavoisier-125 (MIL-125) by a one-pot hydrothermal method [25] as shown in Scheme 1. The highly porous NH_2_-MIL-125(Ti) had a density of hydrophilic active sites/groups (carboxyl, amino, benzene rings, and titanium oxo-clusters). Benefiting from the reactive groups of NH_2_-MIL-125(Ti), FT can be selectively adsorbed from natural water and food samples via hydrogen bonding, π–π interactions, and metal Ti for phosphorus atoms [26]. Then, a non-enzymatic electrochemical sensor was fabricated based on the NH_2_-MIL-125(Ti)/RGO composites for the detection of FT. The NH_2_-MIL-125(Ti)’s strong FT recognition and enrichment capabilities with the outstanding electrical conductivity of RGO contribute to the enhanced detection performance. The fabricated sensor exhibited a high sensitivity with a low detection limit for the detection of FT in agricultural product samples.

## 2. Materials and Methods

### 2.1. Reagents and Chemicals

Ethanol (Et-OH), methanol (Me-OH), N, N-dimethylformamide (DMF), hydrochloric acid (HCl), sulfuric acid (H_2_SO_4_ 98 wt.%), potassium dihydrogen phosphate (KH_2_PO_4_), dipotassium hydrogen phosphate (K_2_HPO_4_), potassium sulphate (K_2_SO_4_), sodium sulphate (Na_2_SO_4_), potassium chloride (KCl), calcium nitrate anhydrous (Ca(NO_3_)_2_), and hydrogen peroxide (H_2_O_2_, 30 wt.%) were received from Sinopharm Chemical Reagent Co., Ltd. (Shanghai, China). Graphene oxide (GO) and titanium isopropoxide (C_12_H_28_O_4_Ti, TPOP 95 wt.%) were purchased from Macklin (Shanghai, China). 2-Aminoterephthalic acid (NH_2_-BDC, 99 wt.%) was obtained from Sigma-Aldrich Corporation (Shanghai, China), and fenitrothion (assay > 95.8%) was supplied by TM-standard (Jiangsu, China). A working solution of 0.1 M phosphate-buffered solution (PBS) containing 80 mM KH_2_PO_4_, 120 mM K_2_HPO_4_, and 100 mM K_2_SO_4_ was used. The ultrapure water (18.20 MΩ cm) was acquired from Milli-Q plus (Millipore, Billerica, MA, USA).

### 2.2. Apparatus and Measurement

Scanning electron microscopy (SEM, Zeiss Gemini 300, Munich, Germany) was used to characterize the surface morphology and microstructure of the materials. The gas chromatograph-mass spectrometer (GC-MS) system consisting of a Thermo Scientific Trace 1300 gas chromatograph coupled to an ISQ 7000 mass spectrometer (Waltham, MA, USA) was utilized for FT quantitation. Fourier transform infrared spectroscopy (FTIR, Nicolet iS5, USA) was used to analyze the functional group status of the materials. Raman spectroscopy (InViaTM Qontor^®^, Renishaw, UK) was used to characterize chemical bonds. The crystalline phases of the samples were identified by X-ray diffraction (XRD, D2 PHASER; Bruker 160 AXS Co., Karlsruhe, Germany). Thermogravimetric analysis (TGA) was performed using a TGA thermal analyzer (TGA/DSC/1100SF, METTLER TOLEDO, USA). The temperature was increased from room temperature to 800 °C at a heating rate of 10 °C/min under the nitrogen atmosphere. All experiments were carried out at a temperature of 22 °C unless otherwise described.

Electrochemical measurements were performed at a CHI660E electrochemical workstation (Chenhua Instruments Co., Ltd., Shanghai, China) in a conventional three-electrode system consisting of a platinum wire auxiliary electrode, an Ag/AgCl (saturated KCl) reference electrode, and a bare glassy carbon electrode (GCE) (3 mm diameter), or a modified GCE as the working electrode.

### 2.3. Synthesis of NH_2_-MIL-125(Ti) and NH_2_-MIL-125(Ti)/RGO

NH_2_-MIL-125 was synthesized using an adapted method [27] in Scheme 1. Firstly, 3.38 mmol titanium isopropoxide and 5.5 mmol 2-aminoterephthalic acid were dissolved in 10 mL of a mixture solution (DMF: Me-OH = 9:1, *v*/*v*). The mixture was then sonicated for half an hour to ensure that the obtained materials were uniformly dispersed. After that, the mixture solution was diverted into a Teflon-lined stainless reaction kettle and the temperature was elevated to 150 °C for 20 h. The suspension was collected by centrifugation (10,000 rpm) after cooling down to room temperature, which was then washed three times with 200 mL of DMF and Me-OH, respectively, to remove the residual complexes. Finally, the suspension was dried in a vacuum at 80 °C for 6 h. NH_2_-MIL-125(Ti)/RGO was synthesized according to a reported procedure [28]. GO (0.005 g) and NH_2_-MIL-125(Ti) (0.05 g) were sonicated and dispersed in 20 mL pure water until the mixture was ultrasonicated to form a homogenous solution. The resulting solution was transferred to a stainless steel reaction kettle lined with Teflon and reacted at 120 °C for 8 h. After cooling to 22 °C, the precipitates were obtained by centrifugation and washed three times with pure water to completely remove the unreacted reagents. Subsequently, the obtained products were dried under a vacuum at 60 °C.

### 2.4. Preparation of NH_2_-MIL-125(Ti)/RGO/GCE

The GCE surface was polished with 0.3 and 0.05 μm alumina powder, then washed for 3 min with deionized water, ethanol, and deionized water. The GCE was then electrochemically treated prior to surface modification, including multiple cyclic voltammetry measurements in 0.5 M H_2_SO_4_ until stable signals were obtained at a potential range from −1.2 to 1.2 V. Then, cyclic voltammetry curves were recorded to test the electrode performance in a solution of 5 mM [Fe(CN)_6_]^3−/4−^ containing 0.1 M KCl with a scan rate of 100 mV/s and a scan range of 0–0.6 V. The electrode can be used only when the peak potential difference in the resulting cyclic voltammogram was below 80 mV. Finally, the electrodes were washed and dried at room temperature. After drying under air, typically, 5 mg of NH_2_-MIL-125(Ti)/RGO was suspended in 5 mL of DI water to form a homogeneous suspension under rigorous ultrasonication. After drying under air, about 5.0 µL of the NH_2_-MIL-125(Ti)/RGO (1 mg/mL) solution was dropped onto the surface of GCE and dried at 22 °C to form a uniform film over the entire electrode surface to obtain NH_2_-MIL-125(Ti)/RGO/GCE. GO/GCE and NH_2_-MIL-125(Ti)/GCE were produced using a similar process. The preparation process and working mechanism of the NH_2_-MIL-125(Ti)/RGO/GCE are shown in Scheme 1.

### 2.5. Electrochemical Measurements

Cyclic voltammetry (CV), electrochemical impedance spectroscopy (EIS), and differential pulse voltammetry (DPV) were used for electrochemical measurements with bare or modified GCE as the working electrodes. The CV response was performed in the potential range from −1.2 to 1.2 V at a scan rate of 100 mV/s in 0.5 mL PBS containing 5 mM [Fe(CN)_6_]^3−/4−^ and 0.1 M KCl. The EIS was performed in an electrolyte containing 5 mM [Fe(CN)_6_]^3−/4−^ and 0.1 M KCl with a range of frequencies from 0.01 to 10^5^ Hz and an amplitude of 5 mV. The DPV measurement was performed over a potential range of −0.3–0.3 V with a scan rate of 100 mV/s and a pulse period of 0.2 s. All measurements were performed in three parallel measurements.

### 2.6. Spiked Sample Preparation

The agricultural products were purchased at a local market in Wuhan (China). The samples were processed according to a reported procedure [20]. It is worth noting that to remove the effects of tannins and other substances, we removed the peels, seeds, and other parts containing interfering substances. A volume of 1 mL of sample extraction solution was diluted with 4 mL of 0.1 M PBS (pH 6.0) solution. As spiked samples, different concentrations of FT were added to diluted extract solutions for FT quantitative detection.

### 2.7. Determination of FT by GC-MS

GC-MS analysis of FT was performed with previous procedures [29]. A 0.5 mL spiked sample with 20 mL acetonitrile was sonicated in an ultrasonic water bath device, and extracted at 60 °C for 40 min. After cooling to room temperature, the mixture was centrifuged at 6000 rpm for 10 min, and then filtered by a 0.22 μm membrane. A volume of 1 mL of the extract in an injection bottle was prepared for GC-MS analysis. The peak areas were determined based on both retention times and mass-to-charge ratios, and the data were acquired in the selected ion monitoring mode. The average integral areas of chromatographic peaks (*n* = 3) were used to calculate the FT concentrations in the real samples, which were then compared with the results of the electrochemical sensor method.

## 3. Results

### 3.1. Characterization of NH_2_-MIL-125(Ti)/RGO

**Figure 1:**
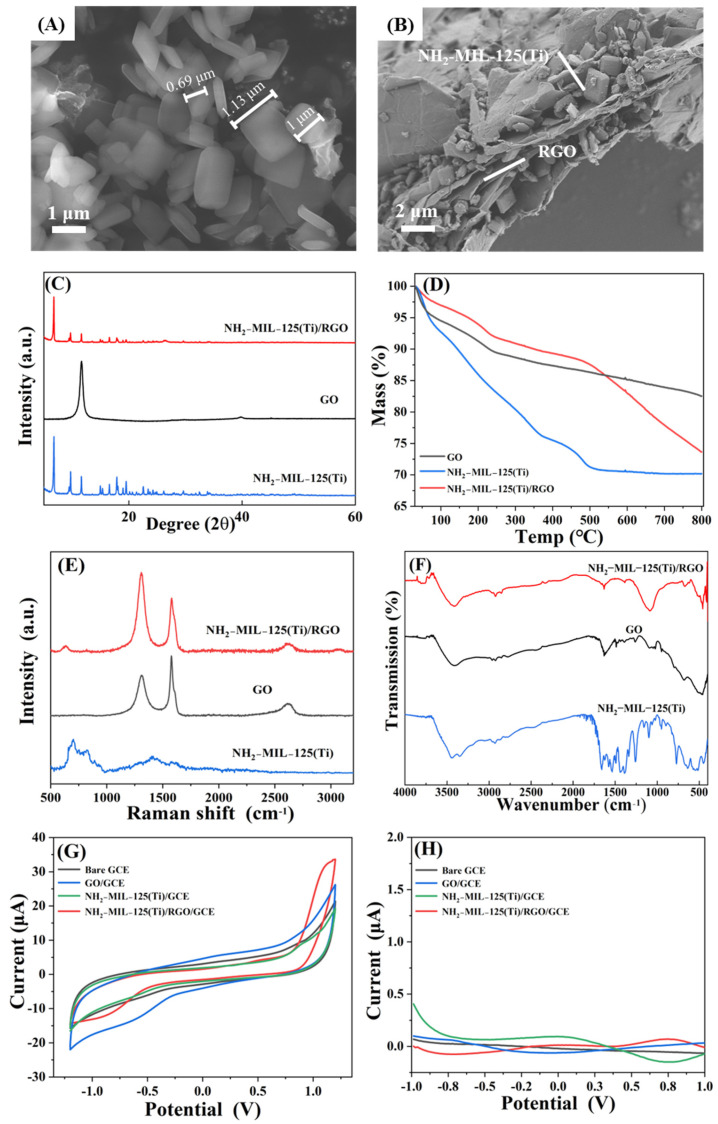
SEM images of (**A**) NH_2_-MIL-125(Ti) and (**B**) NH_2_-MIL-125(Ti)/RGO; (**C**) XRD patterns, (**D**) TGA analysis, (**E**) Raman spectra and (**F**) FTIR spectra of GO, NH_2_-MIL-125(Ti) and NH_2_-MIL-125(Ti)/RGO. (**G**) CV and (**H**) DPV curves in 0.1 M PBS at different modified GCEs.

The SEM images in Figure 1A,B show the morphologies of NH_2_-MIL-125(Ti) and NH_2_-MIL-125(Ti)/RGO. The dispersed NH_2_-MIL-125(Ti) has a nearly circular pie-like structure in Figure 1A and Figure S1A. Figure 1A shows a relatively complete morphology with a smooth and flat outer surface and an average size range from 0.5 to 1.5 μm. In Figure 1B, the synthesized material still has the lamellar structure of RGO, which can provide enough sites for the attachment of NH_2_-MIL-125(Ti). Some highly dispersed grains appeared on the surface of RGO in Figure S1B, and the NH_2_-MIL-125(Ti) particles adhered well in the interlayer of RGO (Figure 1B). Furthermore, the average particle size of (450 ± 200) nm of the NH_2_-MIL-125(Ti) loaded on RGO was smaller than the size of the pure NH_2_-MIL-125(Ti). This may be attributed to the fact that the ultrasonic treatment process increases the number of crystallization centers during the synthesis of the material, which leads to smaller and uniform crystal sizes.

Using XRD, the crystal structure of the composites was investigated. The (101), (200), (211), (222), and (312) planes of the prepared NH_2_-MIL-125(Ti) were represented by the peak values at 6.8°, 9.6°, 11.6°, 16.5°, and 17.9° in Figure 1C, which were in line with the previously reported values [30]. Both the curves of NH_2_-MIL-125(Ti) and NH_2_-MIL-125(Ti)/RGO exhibited distinctive NH_2_-MIL-125(Ti) diffraction peaks. The absence of the 2*θ* = 10.6° diffraction peak at the oxygen-containing groups indicated the successful preparation of RGO from GO [31]. A distinct diffraction peak around 10° corresponding to the (001) diffraction peak of GO was found in the XRD pattern of GO. The XRD pattern of NH_2_-MIL-125(Ti)/RGO was highly similar to NH_2_-MIL-125(Ti), demonstrating that the addition of GO would not destroy the crystal structure of NH_2_-MIL-125(Ti).

The thermal stabilities of NH_2_-MIL-125(Ti)/RGO were estimated by TGA under the nitrogen atmosphere. The TGA curves of GO, NH_2_-MIL-125(Ti), and NH_2_-MIL-125(Ti)/RGO had a decreasing trend below 100 °C in Figure 1D, which might be caused by the evaporation of surface adsorbed unreacted monomer or water molecules. The disintegration of the NH_2_-MIL-125(Ti) framework is completed at around 470 °C [32]. It showed that NH_2_-MIL-125(Ti)/RGO possessed superior thermal stability to that of GO and NH_2_-MIL-125(Ti) until 500 °C.

Raman spectroscopy in Figure 1E was also applied to investigate the NH_2_-MIL-125(Ti)/RGO composition. Two strong characteristic peaks at 1350 and 1580 cm^−1^ were identified in relation to the D and G bands of the GO and NH_2_-MIL-125(Ti)/RGO samples. The stretching vibration of sp^2^ and sp^3^ carbon atoms generated the G and D bands, respectively, which caused defects and disorders [28]. The RGO structure’s disorder and flaws were indicated by the intensity ratio of the D/G bands (*I_D_*/*I_G_*) [31], where the ratios for GO and NH_2_-MIL-125(Ti)/RGO were 0.69 and 1.48, respectively. The rise in the *I_D_*/*I_G_* value of NH_2_-MIL-125(Ti)/RGO might be due to the elimination of oxygen−containing groups from GO by thermally combining with NH_2_-MIL-125(Ti). This contributed to the successful attachment of NH_2_-MIL-125(Ti) to the RGO surface layer.

The FTIR spectrum of NH_2_-MIL-125(Ti) showed a -NH_2_ stretching vibration absorption peak from the organic ligand of NH_2_-MIL-125(Ti) near 3400 cm^−1^ in Figure 1F. The absorption at 400–800 cm^−1^ was derived from the O−Ti−O stretching vibration [33]. The peaks near 1500 and 1600 cm^−1^ resulted from the asymmetric stretching vibration of C=O, whilst those at 1400 and 1440 cm^−1^ were from C=O symmetric stretching vibrations [28]. The absorption peaks of NH_2_-MIL-125(Ti)/RGO near 750, 1700, and 3400 cm^−1^ indicated the successful synthesis of NH_2_-MIL-125(Ti)/RGO.

CV and DPV were employed to assess the electrochemical performance of NH_2_-MIL-125(Ti)/RGO in 0.1 M PBS. In Figure 1G, it is shown that no evident peak appeared in the CVs for GCE after the modification with GO, NH_2_-MIL-125(Ti), and NH_2_-MIL-125(Ti)/RGO. It revealed that the material had stable performance and no interfering electrochemical signal under electrochemical detection conditions. The results were in agreement with the DPV results in Figure 1H. The above characterization results indicated that NH_2_-MIL-125(Ti)/RGO was successfully synthesized, displayed excellent properties from both NH_2_-MIL-125(Ti) and GO, and could be used for subsequent studies.

### 3.2. Electrochemical Investigation and Determination of FT

#### 3.2.1. Electrochemical Behavior of the Modified Electrodes

**Figure 2:**
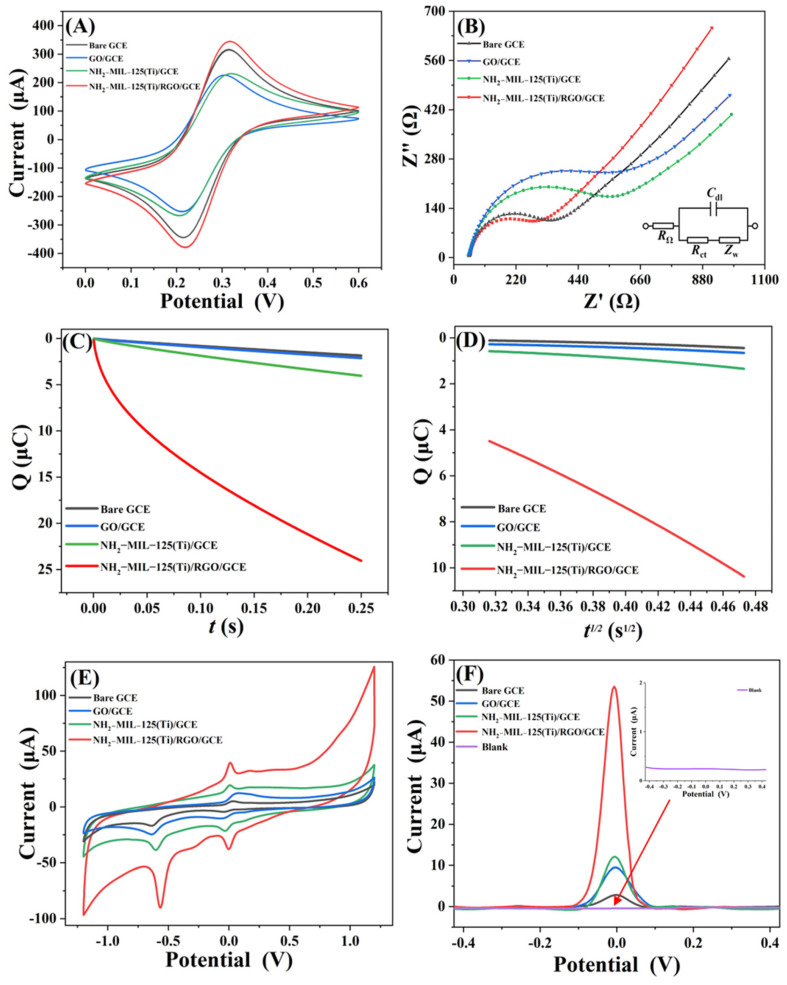
(**A**) CVs and (**B**) EIS of different GCEs in 5 mM Fe(CN)_6_^3−/4−^ containing 0.1 M KCl; (**C**) Plot of Q~*t* and (**D**) Q~*t*^1/2^ curves of different GCEs; (**E**) CV and (**F**) DPV responses of different GCEs for 3.6 mM FT in 0.1 M PBS.

The electrochemical behavior of the different modified GCEs was explored by CV. As shown in Figure 2A, CVs of different modified GCEs in a 5 mM Fe(CN)_6_^3−/4−^ solution (with 0.1 M KCl) were observed, revealing the electrochemical reaction of ferricyanide was a quasi-reversible process. The peak potential difference (∆Ep) of bare GCE, GO/GCE, NH_2_-MIL-125(Ti)/GCE, and NH_2_-MIL-125(Ti)/RGO/GCE were equal to 0.102, 0.110, 0.098, and 0.094 V, respectively. Due to its superior electron conductivity and substantial surface area compared to the other electrodes, NH_2_-MIL-125(Ti)/RGO/GCE exhibited the highest redox peak current and the lowest ∆Ep [28].

EIS can further characterize the electron transfer capabilities of the electrode and provide information about the surface impedance change [34]. The diameter of the semicircle on the Nyquist plots produced for different GCEs corresponds to the respective charge-transfer resistance (*R*_ct_). In Figure 2B, the *R*_ct_ were 374 Ω, 803 Ω, 607 Ω, and 326 Ω for the bare GCE, the GO/GCE, NH_2_-MIL-125(Ti)/GCE, and NH_2_-MIL-125(Ti)/RGO/GCE, respectively. The low *R*_ct_ of NH_2_-MIL-125(Ti)/RGO/GCE clearly showed excellent conductivity, favorable electron transfer rate, and large specific surface area of the modified GCE, indicating that the introduction of RGO had a significant effect on the enhancement of the conductivity of modified electrodes.

#### 3.2.2. Chronocoulometric Behavior and Reaction Mechanism

Chronocoulometry was employed for the determination of some kinetic parameters and reaction mechanisms. The effective surface areas were studied for different GCEs in 1 mM Fe(CN)_6_^3−/4−^ containing 0.1 M KCl. The relationships between charge (*Q*) and potential pulse width (*t*^1/2^) were different for bare GCE, GO/GCE, NH_2_-MIL-125(Ti)/GCE, and NH_2_-MIL-125(Ti)/RGO/GCE in Figure 2D. According to Anson’s equation [35]:

*Q*(*t*) = 2*ncFAD*^1/2^*t*^1/2^π^−1/2^ + Q_dl_ + Q_ads_ (1)

where *n*, *c*, *F*, *A*, *D, Q*_dl_, and *Q*_ads_ were the transferred electron number, the concentration of substrate, Faraday’s constant, the effective surface area of the working electrodes, the diffusion coefficient, the double layer charge, and the Faradic charge, respectively. The effective surface areas of the bare GCE and the GCEs modified with GO, NH_2_-MIL-125(Ti), and NH_2_-MIL-125(Ti)/RGO were calculated to be 0.027, 0.031, 0.063, and 0.473 cm^2^, respectively. The results showed that the addition of NH_2_-MIL-125(Ti)/RGO greatly enhanced the effective surface area of the electrode.

#### 3.2.3. Feasibility Analysis of Electrochemical Sensor

The electrochemical behaviors of the different GCEs toward 360 μM FT were investigated in 0.1 M PBS. When the GCE was modified with NH_2_-MIL-125(Ti)/RGO in Figure 2E, the current of FT increased dramatically to around 0.08 V, but the potential change was not apparent. There was a slight shift of GO and NH_2_-MIL-125(Ti)-modified GCE toward the negative charge direction at about 0.08 V peak potential. In addition, NH_2_-MIL-125(Ti)/RGO/GCE showed a distinct and strong reduction peak at 0.08 V, and its reduction peak currents were 2.9, 3.1, and 5.7 times higher than that of GO/GCE, NH_2_-MIL-125(Ti)/GCE, and bare GCE, respectively. A weak reduction peak I at −0.68 V with reduced signal intensity was observed for the bare GCE, which was responsible for the electrochemical reduction of the nitro in FT to phenylhydroxylamine [36]. The evaluation of the smaller decrease peak I of the reaction indicated that the electrochemical reaction of FT exhibited irreversible activity. Throughout the reverse scan (oxidation peak I and reduction peak II almost both at 0.08 V), the oxidation of benzylamine to nitroso produced redox peaks, which was followed by a sequential reversible reduction transfer process of 2e^−^ and 2H^+^. According to the results and the reference [36], Figure S2 depicted the possible total catalytic reduction mechanism of FT on the modified electrode.

### 3.3. Optimization of Experimental Conditions

#### 3.3.1. Effect of pH

**Figure 3:**
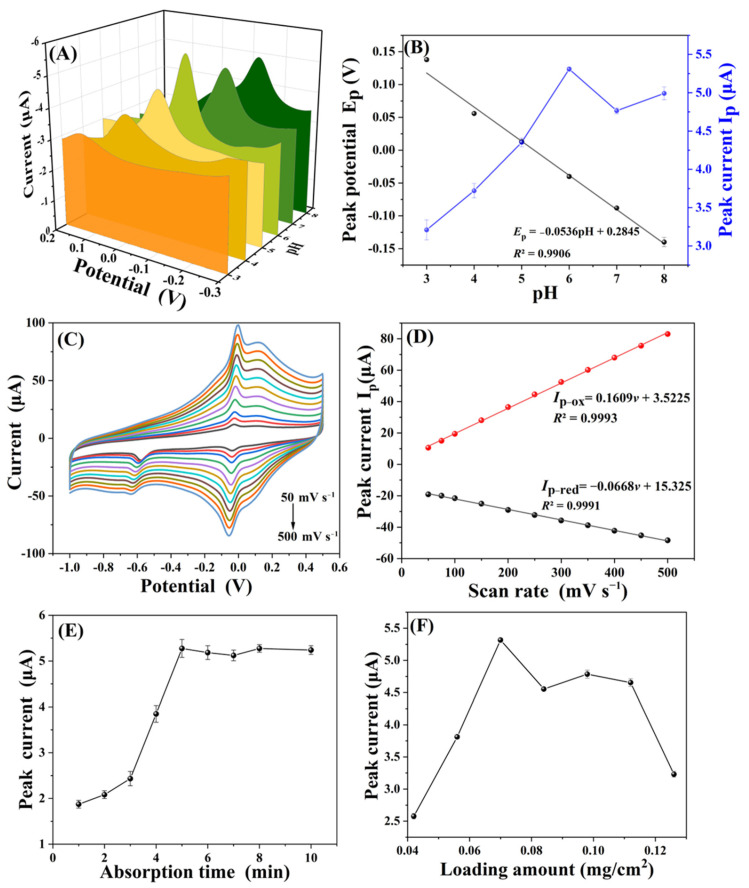
(**A**) DPVs of 360 μM FT in different pH; (**B**) Variation of peak potential and current of 360 μM FT with pH; (**C**) CVs of NH_2_-MIL-125(Ti)/RGO/GCE in PBS containing 360 μM of FT at different scan rates: (50, 75, 100, 150, 200, 250, 300, 350, 400, 450, and 500 mV/s); (**D**) Linear relationship between the peak current and scan rate; (**E**) Effect of absorption time of FT solution (360 μM) on the reduction peak current; (**F**) Effect of loading amount of NH_2_-MIL-125(Ti)/RGO on the reduction peak current of 360 μM FT.

Since the potentials of the oxidation and reduction peaks of FT depend on the pH of the solution, and protons are involved in the reduction of FT*_ox_* to FT*_red_* [37], the variation patterns of the peak current and potential of FT with pH were discussed in detail. The effect of pH on the electrochemical performance of 360 μM FT at the NH_2_-MIL-125(Ti)/RGO/GCE was evaluated by DPV in the pH range of 3.0–8.0. It was found that the peak potential tilted from positive to low negative values and the peak current showed an increasing trend as the pH of the buffer solution increased (Figure 3A), which was derived from the involvement of H^+^ in the electrode interactions [38]. The peak current tended to increase with pH, attaining a maximum at pH 6.0 in Figure 3B. In addition, the linear equation between peak potential and pH was expressed as *E*_p_(V) = −0.0536 pH + 0.2845 (*R*^2^ = 0.9906). The slope’s absolute value was approximately equal to the universal theoretical value of 0.059 V/pH [39], showing that the oxidation of FT on NH_2_-MIL-125(Ti)/RGO/GCE was a double proton and electron process in Figure S2, where the number of protons was equal to the number of electron transfers in the electrochemical oxidation reaction.

#### 3.3.2. Effect of Scan Rate

The effect of the sweep rate on the peak potential and current of FT was determined in 0.1 M PBS (pH 6.0) and in the scan range of 50–500 mV/s using CV on the NH_2_-MIL-125(Ti)/RGO modified GCE (Figure 3C). The equation and correlation coefficient of redox peak current versus scan rate in Figure 3D was *I*_p-ox_ = 0.1609*v* + 3.5225, *R*^2^ = 0.9993 and *I*_p-red_ = −0.0668*v* + 15.3250, *R*^2^ = 0.9991, respectively. It also showed that the FT oxidation peak potential of the modified GCE surface shifted slightly toward the positive potential and the reduction peak potential shifted slightly toward the negative potential as the scanning speed increased, which was regulated by diffusion.

#### 3.3.3. Effect of Accumulation Time and Loading Amount

Since the porous material NH_2_-MIL-125(Ti)/RGO was adsorptive and the saturation adsorption of FT required a certain time, the adsorption time of FT was optimized. The impact of adsorption time on the peak current response of FT on the NH_2_-MIL-125(Ti)/RGO-modified GCE was studied in Figure 3E. With prolonged absorption time, the peak current of FT raised and reached its maximum at 5 min, and there was no obvious change thereafter. Therefore, the optimal deposition duration for FT detection was determined to be 5 min.

In Figure 3F, the peak current rose when a higher amount of NH_2_-MIL-125(Ti)/RGO was immobilized. When the loading amount was 0.07 mg/cm^2^, the peak current of FT reached its maximum value. Afterward, the peak current was gradually reduced with a further increase in the loading amount of the composites. The possible reason might be that the thick material prevented the transfer of electrons from the electrode surface to the material after it has collected on the electrode surface [36]. Therefore, 0.07 mg/cm^2^ of NH_2_-MIL-125(Ti)/RGO was employed for the follow-up experiments.

### 3.4. Analytical Performance of the Electrochemical Sensor

#### 3.4.1. Sensitivity of the Fabricated Sensor for FT Detection

**Figure 4:**
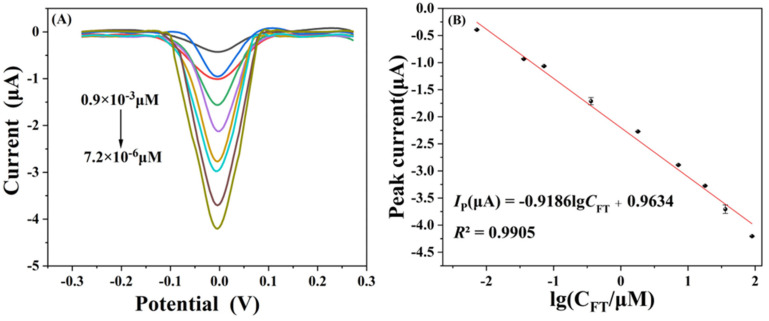
(**A**) Differential pulse voltammogram of different FT concentrations at NH_2_-MIL-125(Ti)/RGO/GCE. (**B**) Calibration curve between peak current (µA) and the logarithm of FT concentrations (μM) (*n* = 3).

DPV was adopted for the quantitative determination of FT. The DPV curves of different concentrations of FT were presented in Figure 4A at NH_2_-MIL-125(Ti)/RGO/GCE under optimized conditions. The reduction peak current of DPV at around −0.04 V increased with increased FT concentrations in Figure 4A. The linear relationship between peaks current (*I*/µA) and FT concentration (µM) was expressed as *I_p_* (µA) = −0.9186 × lg*C*_FT_ + 0.9634 (*R*^2^ = 0.9905) in the range of 0.072–18 μM (Figure 4B). The limit of detection (LOD, 3 × *S/N*) and the limit of quantification (LOQ, 10 × *S/N*)) were calculated, where *S* was the standard deviation of a blank solution and *N* was the slope of the calibration curve. The estimated LOD and LOQ were 0.0338 μM and 0.0427 μM, respectively. It showed that the developed sensor had excellent sensitivity to FT with a wide linear range. Some reported electrochemical methods for FT determination were summarized in Table S1 [40,41,42,43,44,45,46,47]. Compared with those reported methods, the developed electrochemical method has comparable or even better performance in the detection range and LOD. It indicates that the FT can be accurately detected using the electrode modified with NH_2_-MIL-125(Ti)/RGO. It could be attributed to the effective affinity effect of the titanium atom of NH_2_-MIL-125(Ti) for the phosphorus atom in FT [26] and the good electron transfer efficiency of RGO.

#### 3.4.2. Reproducibility, Repeatability, Stability, and Selectivity

The reproducibility of the developed electrochemical sensor was accessed in 0.1 M PBS (pH 6.0) by five sensors prepared as in Section 2.5. The average current response of 360 μM FT on five sensors was (5.43 ± 0.05) μA with an relative standard deviation (RSD) of 4.24% (*n* = 3) in Figure S3A, indicating that the developed sensor had outstanding reproducibility. In addition, the repeatability of the NH_2_-MIL-125(Ti)/RGO/GCE was assessed by measuring the DPVs of 360 μM FT for 10 consecutive times in Figure S3B. The average peak current was (5.43 ± 0.21) μA with an RSD of 3.94%, revealing that the developed sensor had good repeatability. Moreover, the stability was studied through the measurements of 360 μM FT for 15 days in Figure S3C. It demonstrated that the NH_2_-MIL-125(Ti)/RGO-based sensor still retained more than 95% of its initial current after 15 days, demonstrating that the designed sensor had good long-term storage stability.

Selectivity was studied by evaluating the effect of some interfering compounds such as Na^+^, K^+^, Cl^−^, Ca^2+^, NO_3_^−^, SO_4_^2−^ as well as other organophosphate pesticides (carbofuran, trans-heptachlor epoxide, malathion, chlorpyrifos) on the peak currents of FT at the developed sensor. The relative response currents of 3.6 mM interfering compounds were all within 1.11% of that of 360 μM FT in Figure S4. Moreover, the mixed interfering compounds insignificantly affect the signal obtained from FT oxidation. The results indicated that the designed sensor could specifically detect FT independent of the common interfering compounds.

### 3.5. Sensor Performance in Extracts from Agricultural Product Samples

Common agricultural products were selected as real samples to assess the applicability and feasibility of the fabricated sensor. Complex food matrices could interfere with the detection results of the sensor. For example, tannic acid, a hydrolyzed polyphenol compound, is abundant in different types of fruits and vegetables. Since substances such as tannic acid can interfere with the electrochemical detection process [48], tannic acid needs to be removed as much as possible in the pretreatment process. After pretreatment, the sample extract solution was transferred to 0.1 M PBS, then added to different concentrations of FT for DPV measurement in an electrochemical detection system. The mean recoveries with the electrochemical method were 94.21–103.44%, while the mean recoveries for the GC-MS method were 96.85–102.67% (Table 1). The results for real sample analyses (three repeated measurements) indicated great recovery and repeatability and were in good agreement with the results obtained by the reference method (GC-MS). The results recommended that the developed sensor could be applied successfully to detect FT in agricultural product samples.

## 4. Conclusions

To address the restriction of enzyme inhibition-based methods in applications, a simple and sensitive non-enzymatic electrochemical sensor has been successfully developed to detect FT by utilizing NH_2_-MIL-125(Ti)/RGO composites. The NH_2_-MIL-125(Ti)/RGO composites were synthesized by a one-pot method with simple preparation and stable properties. The composite combined the high surface area and superior adsorption capacity of NH_2_-MIL-125(Ti) with the efficient electron transport capacity of RGO. At the same time, the strong affinity of NH_2_-MIL-125(Ti) for phosphorus atoms in FT has been fully utilized for the enrichment of FT. These advantages synergistically enhanced the detection performance of the constructed electrochemical sensor. The quantitative analysis of FT over the range of 0.072–18 μM dynamic variation was realized based on the sensor. The LOD and LOQ were 0.0338 μM and 0.0427 μM, respectively. Moreover, the developed electrochemical method had comparable or even better performance in detection range and LOD. The proposed method exhibited good reproducibility, repeatability, selectivity, and acceptable recoveries in real samples. This work provides a promising approach for the development of non-enzymatic electrochemical sensors for the rapid detection of FT in agricultural products.

## Figures and Tables

**Table 1:** The recovery was obtained for the analysis of agricultural product samples (*n* = 3).

Sample		FT Added (μM)	FT Found (Mean ± SD) (μM)	Recovery (%)	RSD (%)	GC-MS Validation (Mean ± SD) (μM)	Recovery ^a^ (%)	RSD (%) ^b^
Cereals	Rice flour	0.36	0.36 ± 0.04	102.24	2.13	0.36 ± 0.01	99.35	1.98
		1.8	1.81 ± 0.03	100.47	1.22	1.81 ± 0.01	100.52	1.65
		3.6	3.59 ± 0.04	99.78	1.87	3.61 ± 0.04	100.19	1.02
	Wheat flour	0.36	0.35 ± 0.07	98.37	2.45	0.35 ± 0.01	96.85	2.04
		1.8	1.82 ± 0.04	101.79	2.34	1.80 ± 0.02	100.24	1.75
		3.6	3.58 ± 0.06	99.70	1.94	3.62 ± 0.07	100.66	2.04
Vegetables	Cucumber	0.36	0.36 ± 0.01	103.44	2.81	0.36 ± 0.01	101.30	1.89
		1.8	1.77 ± 0.08	98.07	1.98	1.78 ± 0.03	99.65	1.53
		3.6	3.68 ± 0.01	102.33	1.57	3.63 ± 0.06	100.81	1.49
	Tomato	0.36	0.34 ± 0.01	94.21	3.74	0.36 ± 0.01	100.83	2.34
		1.8	1.83 ± 0.02	101.56	2.94	1.81 ± 0.03	100.70	1.58
		3.6	3.53 ± 0.10	97.93	1.58	3.62 ± 0.07	100.62	1.30
Fruits	Grape	0.36	0.35 ± 0.01	97.22	2.91	0.37 ± 0.01	102.67	1.26
		1.8	1.70 ± 0.07	94.28	3.75	1.81 ± 0.02	100.81	1.50
		3.6	3.67 ± 0.07	102.01	1.95	3.65 ± 0.04	101.43	0.99
	Peach	0.36	0.35 ± 0.01	96.67	2.21	0.35 ± 0.01	98.22	1.91
		1.8	1.77 ± 0.01	98.20	1.74	1.82 ± 0.01	100.69	1.48
		3.6	3.70 ± 0.02	102.06	1.69	3.64 ± 0.02	100.58	1.27

^a^: Recovery (%) = FT _added_/FT _found_ ∗ 100%; ^b^: RSD (%) = Standard Deviation/Mean ∗ 100%.

## Data Availability

Data is contained within the article or Supplementary Materials.

## Supplementary Materials

The following supporting information can be downloaded at: https://www.mdpi.com/article/10.3390/foods12071534/s1, Figure S1: SEM images of (A) NH_2_-MIL-125 and (B) NH_2_-MIL-125(Ti)/RGO; Figure S2: The electrochemical mechanism of the FT at the RGO/NH_2_-MIL-125(Ti)/GCE; Figure S3: (A) Reproducibility, (B) Repeatability, and (C) Stability measurements of 360 μM FT using the NH_2_-MIL-125(Ti)/RGO/GCE in 0.1 M PBS (pH 6.0); Figure S4: Selectivity of the developed electrochemical sensor for FT against other interferents; Table S1: Comparative study of performances of different electrochemical sensors for detection of FT.
