# Supplementary material for: NH2-MIL-125(Ti)/Reduced Graphene Oxide Enhanced Electrochemical Detection of Fenitrothion in Agricultural Products
Source: Foods. 2023 Apr 4;12(7):1534. doi: 10.3390/foods12071534 (PMC10093892; doi:10.3390/foods12071534)
Supplement: Supplementary file 1 [file foods-12-01534-s001.zip › foods-2297841-supplementary.pdf]

foods

Electronic Supplementary Material For

NH_2-MIL-125(Ti)/Reduced Graphene Oxide Enhanced Electrochemical Detection of Fenitrothion in Agricultural Products

Zaixi Shu [1,†], Yue Zou [1,†], Xuyue Wu [1], Qi Zhang [2], Yafang Shen [3], Anhong Xiao [1], Shuo Duan [1,4], Fuwei Pi [1,5], Xiaodan Liu [1], Jiahua Wang [1] and Huang Dai [1,4,*]

[1] College of Food Science and Engineering, Wuhan Polytechnic University, Wuhan 430023, China
[2] School of Grain Science and Technology, Jiangsu University of Science and Technology, Zhenjiang 212004, China
[3] Zhejiang Institute of Freshwater Fisheries, Huzhou 313001, China
[4] Food Safety Research Center, Key Research Institute of Humanities and Social Sciences of Hubei Province, Wuhan 430023, China
[5] School of Food Science and Technology, Jiangnan University, Wuxi 214122, China
* Correspondence: huangdai9@126.com; Tel.: +86 27 83924790
† These authors contributed equally to this work.

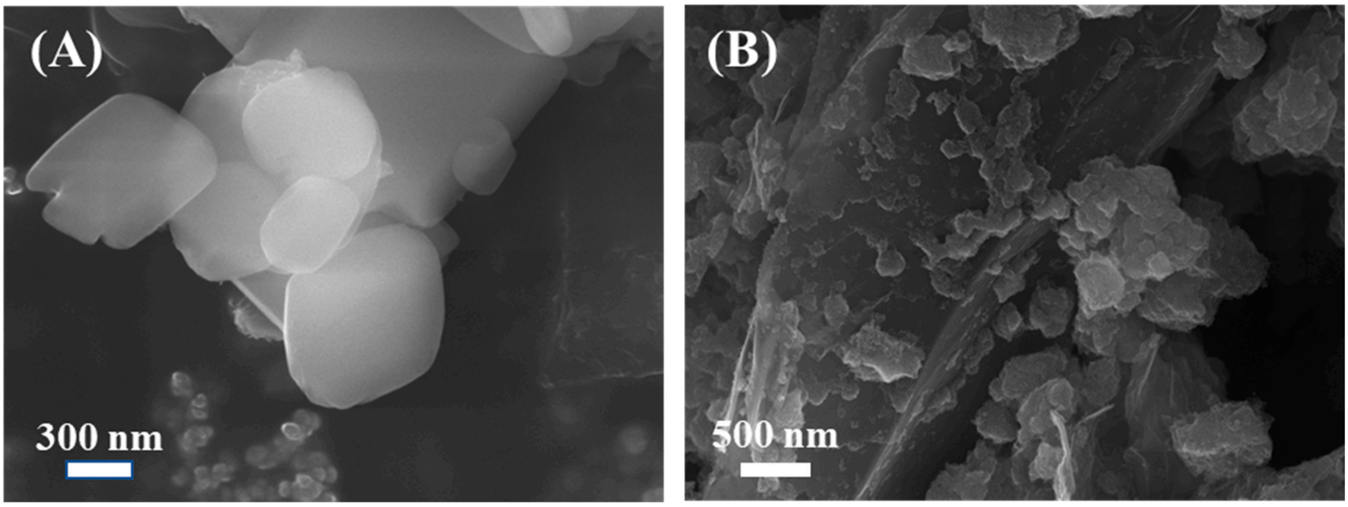

Figure S1. SEM images of (A) NH_2−MIL−125(Ti) and (B) NH_2−MIL−125(Ti)/RGO

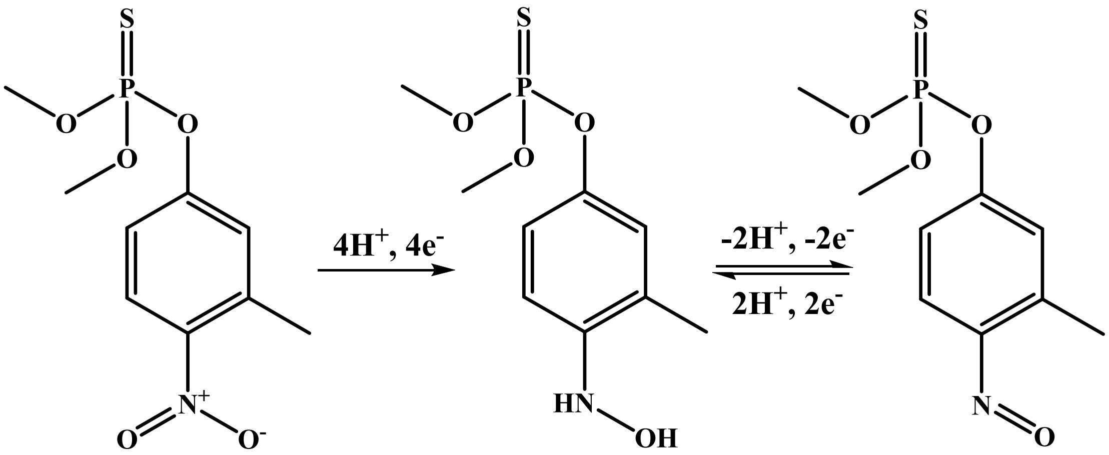

Figure S2. The electrochemical mechanism of the FT at the NH_2–MIL–125(Ti)/RGO/GCE

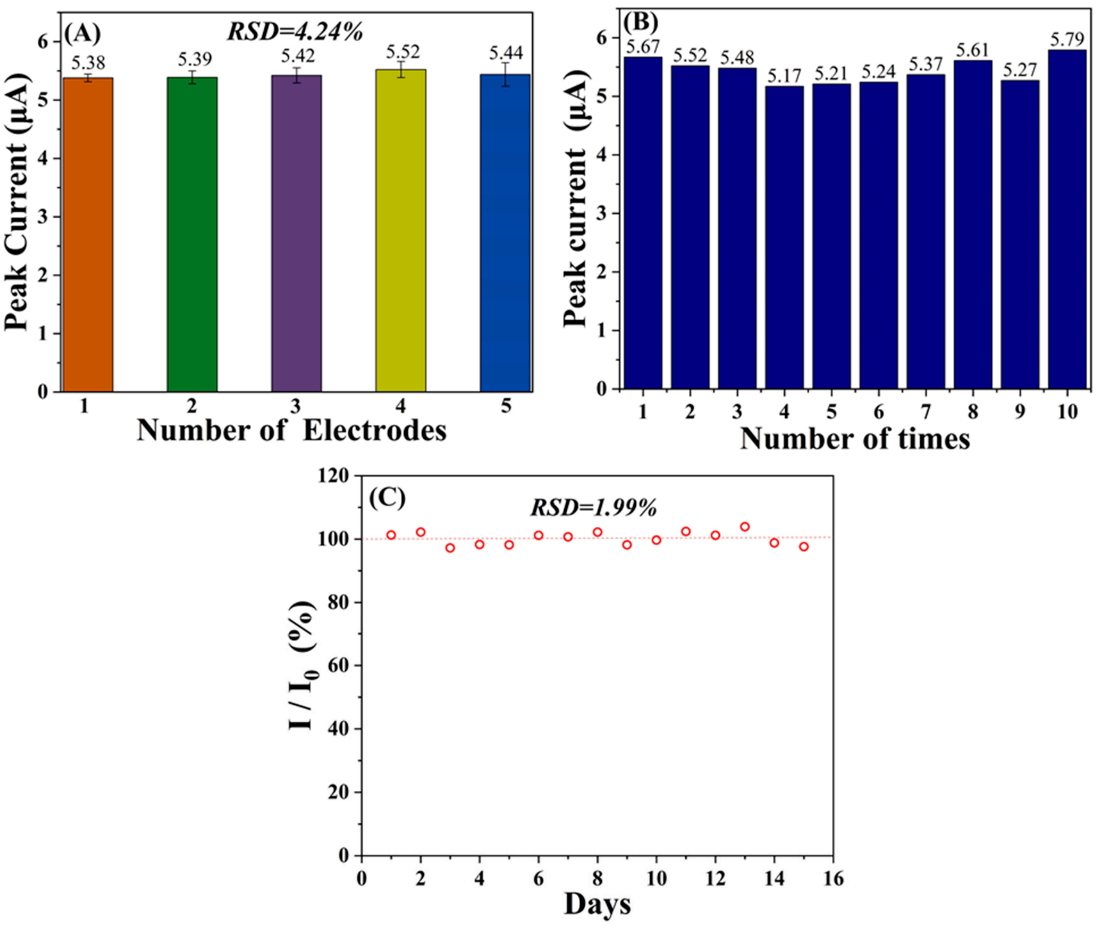

Figure S3. (A) Reproducibility, (B) Repeatability and (C) Stability measurements of 360 μM FT using the NH_2−MIL−125(Ti)/RGO/GCE in 0.1 M PBS (pH 6.0).

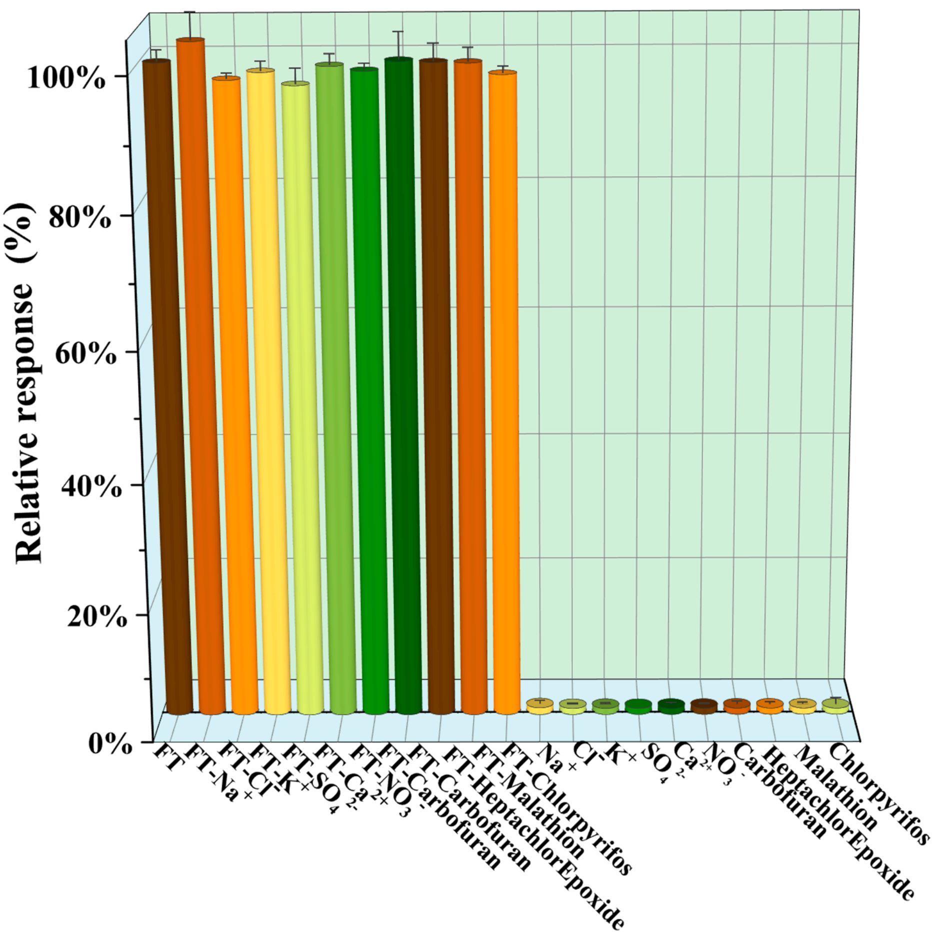

Figure S4. Selectivity of the developed electrochemical sensor for FT against other interferents. (from left to right: FT, FT−Na^+, FT−Cl^-, FT−K^+, FT−SO_4^{2-}, FT−Ca^{2+}, FT−NO_3^-, FT−Carbofuran, FT−Trans−Heptachlor Epoxide, FT−Malathion, FT−Chlorpyrifos, Na^+, Cl^-, K^+, SO_4^{2-}, Ca^{2+}, NO_3^-, Carbofuran, Trans−Heptachlor Epoxide, Malathion, Chlorpyrifos). The concentration is 360 μM for FT and 3.6 mM for other interferents.The error lines indicates the mean standard error of the three measurements. The error bars represent average standard error for three measurements.

Table S1 Comparative study of performances of different electrochemical sensors for detection of FT

No.	Materials used	Sample	Linear Ranges (μM)	LOD (μM)	Ref.
1	Poly(purpald)−PGE	apple juice, water	25–200	17.39	[1]
2	TiO_2/Nafion/GCE	water	0.2–4	0.0866	[2]
3	PHA/mPEG/SPE	tap water	1–10	0.61	[3]
4	MWCNTs−GCE	river water	0.2–60	0.08	[4]
5	Pretreated GCE	tap water, human urine	0.4–50	0.078	[5]
6	Ni(II) MIP−SPCE	forest matrices	3.00–100	0.08	[6]
7	CoPc/GCE	/	1.20–42.0	0.46	[7]
8	SiO_2/CNTs/RuPc	orange juice	3–66	0.162	[8]
9	SWCNH−ZE/GCE	natural water, orange juice	0.99–12	0.012	[9]
10	IL@$CoFe_2O_4$NPs@MWCNTs	fruit extract	0.02–160	0.0135	[10]
11	NH_2−MIL−125(Ti)/RGO	agricultural products extract (rice, wheat, grape, peach, cucumber, tomato)	0.072–18	0.0338	This work

CV: cyclic voltammetry; EIS: electrochemical impedance spectroscopy; DPV: differential pulse voltammetry; PGE: pencil graphite electrode; GR: graphene; GCE: glassy carbon electrode; MWCNTs: multi−walled carbon nanotubes; RGO: reduced graphene oxide; SPCE: Screen Printed Carbon Electrodes; AuNPs: gold nanoparticles; CNTs: carbon nanotubes; RuPc: ruthenium phthalocyanine; ZE: zein; IL: ionic liquid; $CoFe_2O_4$NPs: cobalt ferrite nanoparticles; mPEG: poly(ethylene glycol) methyl ether; PHA: polyhydroxyalkanoates; CoPc: Cobalt phthalocyanine.

References

1. Soysal M. Voltammetric determination of fenitrothion based on pencil graphite electrode modified with poly(Purpald((R))). *Chem. Pap.* **2019**, 73(7), 1785-1794.
2. Kumaravel A.; Chandrasekaran M. A biocompatible nano TiO_2/nafion composite modified glassy carbon electrode for the detection of fenitrothion. *J. Electroanal. Chem.* **2011**, 650(2), 163-170.
3. Sgobbi L.F.; Machado S.A.S. Functionalized polyacrylamide as an acetylcholinesterase-inspired biomimetic device for electrochemical sensing of organophosphorus pesticides. *Biosens. Bioelectron.* **2018**, 100, 290-297.
4. Salehzadeh H.; Ebrahimi M.; Nematollahi D.; Salarian A.A. Electrochemical study of fenitrothion and bifenox and their simultaneous determination using multiwalled carbon nanotube modified glassy carbon electrode. *J. Electroanal. Chem.* **2016**, 767, 188-194.
5. Geremedhin W.; Amare M.; Admassie S. Electrochemically pretreated glassy carbon electrode for electrochemical detection of fenitrothion in tap water and human urine. *Electrochim. Acta* **2013**, 87, 749-755.
6. Pellicer C.; Gomez-Caballero A.; Unceta N.; Goicolea M.A.; Barrio R.J. Using a portable device based on a screen-printed sensor modified with a molecularly imprinted polymer for the determination of the insecticide fenitrothion in forest samples. *Anal. Methods* **2010**, 2(9), 1280-1285.
7. Akyüz D.; Keleş T.; Biyiklioglu Z.; Koca A. Electrochemical pesticide sensors based on electropolymerized metallophthalocyanines. *J. Electroanal. Chem.* **2017**, 804, 53-63.
8. Canevari T.C.; Prado T.M.; Cincotto F.H.; Machado S.A.S. Immobilization of ruthenium phthalocyanine on silica-coated multi-wall partially oriented carbon nanotubes: Electrochemical detection of fenitrothion pesticide. *Mater. Res. Bull.* **2016**, 76, 41-47.
9. Itkes M.P.M.; De Oliveira G.G.; Silva T.A.; Fatibello O.; Janegitz B.C. Voltammetric sensing of fenitrothion in natural water and orange juice samples using a single-walled carbon nanohorns and zein modified sensor. *J. Electroanal. Chem.* **2019**, 840, 21-26.
10. Kilele J.C.; Chokkareddy R.; Redhi G.G. Ultra-sensitive electrochemical sensor for fenitrothion pesticide residues in fruit samples using IL@$CoFe_2O_4$NPs@MWCNTs nanocomposite. *Microchem. J.* **2021**, 164, 106012.
